# The Impact of Selected Eutectic Solvents on the Volatile Composition of *Citrus lemon* Essential Oil

**DOI:** 10.3390/ma17215288

**Published:** 2024-10-30

**Authors:** Giacomo Luigi Petretto, Andrea Mele, Giorgio Pintore, Alberto Mannu

**Affiliations:** 1 Department of Medicine, Surgery and Farmacy, University of Sassari, 07100 Sassari, Italy; pintore@uniss.it; 2 Department of Chemistry, Materials and Chemical Engineering “G. Natta”, Politecnico di Milano, Piazza L. da Vinci 32, 20133 Milano, Italy; andrea.mele@polimi.it

**Keywords:** *Citrus* essential oil, controlled release, choline chloride, methyltriphenylphosphonium bromide, EcoScale

## Abstract

The development of new materials for the controlled release of molecules represents a topic of primary importance in medicine, as well as in food science. In recent years, eutectic solvents have been applied as releasing media due to their improved capacity to interact with specific molecules, offering a broad range of tunability. Nevertheless, their application in essential oil dissolution are rare and more data are needed to develop new generations of effective systems. Herein, three eutectic systems, respectively, composed of choline chloride and ethylene glycol (1:2 molar ratio), methyltriphenylphosphonium bromide and ethylene glycol (molar ratio 1:5), and choline chloride and glycerol (molar ratio 1:1.5) were tested as materials for the controlled release of an essential oil derived from *Citrus lemon* leaves. Through static headspace fractionation, followed by gas chromatographic analysis, the performances of the three systems were assessed. The specific composition of DESs was pivotal in determining the releasing polar molecules as aldehydes and alcohols. A sustainability ranking based on the EcoScale tool highlighted the superior characteristics of the choline chloride–glycerol DES.

## 1. Introduction

Among the wide range of essential oil (EO) applications, their potential as antimicrobial agents has been extensively studied [1]. The antimicrobial activity of essential oils (EOs) is closely linked to their chemical composition, often exhibiting stronger effects than individual components due to synergistic interactions [2]. In particular, *Citrus limon* has shown significant antimicrobial effects, mainly attributed to the high concentration of citral (a mixture of the two isomers neral and geranial) in the EO.

*Citrus* leaves are known as valuable sources of citrus EO [3,4,5], which is obtained through steam distillation [6]. Its chemical profile is characterized by a mixture of monoterpenes, sesquiterpenes, alcohols, aldehydes, and esters [3,7,8]. *Citrus* leaf EO is usually rich in limonene [9], geranial, neral, citronellal, sabinene, linalool, (E)-ocimene, geraniol, geranyl acetate, linalyl acetate, alpha-terpineol, and myrcene [9,10,11]. This complex mixture of organic compounds shows wide biological activity against different pathogens. The employment of *Citrus* EOs as antimicrobial [12,13], analgesic [3] insecticidal [14] anti-leishmanial [4], and antioxidant agents [5,9,10] was reported. The biological activity of essential oils has been extensively studied in the liquid phase [15], where it is possible to determine the minimal inhibitory concentration. Moreover, several authors have recently reported studies on the biological activity of essential oils in the vapor phase of food matrices [16].

In this context, the designing of suitable systems for the controlled release of *Citrus* leaf EO has gained attention. The general addition of EOs to a solid–liquid matrix, or their deposition by filmcasting, represent the most employed solutions for exploiting their antioxidant and antibacterial properties in food packaging [17]. Most of the materials able to include EOs are polymers as polypropylene, polyethylene, terephthalate. Also, some packages based on proteins, cellulose, and chitosan were reported [17]. The application of such materials in smart food packaging is limited by their low adaptability to different chemical groups. However, the ability to design systems that can adapt to specific volatile organic compounds (VOCs) and easily and effectively adjust their interactions, if necessary, can be achieved using eutectic mixtures.

In this context, the application of eutectic mixtures in this specific field remains largely unexplored. Eutectic systems, such as deep eutectic solvents (DESs), offer a promising alternative for the dissolution and controlled release of organic molecule mixtures, such as citrus leaf essential oil (EO). DESs are typically binary or ternary eutectic systems composed of specific combinations of hydrogen bond donors (HBDs) and hydrogen bond acceptors (HBAs), which exhibit a melting point significantly lower than the theoretical value. Since the first report by Abbott and co-workers on the eutectic properties of mixtures between choline chloride and quaternary ammonium salts [18], a growing number of DESs and their analogs have been designed and documented [19]. A general summary of the types of DESs is provided in Table 1.

Regarding the many applications of DESs, their employment as release agents for active formulations is well known [20]. The application of DESs for dissolving citrus leaf EO and studying their release kinetics can help identify which combinations of HBAs and HBDs are most suitable for practical applications.

In the present research paper, three DESs were tested as materials for the controlled release of *Citrus limon* essential oil obtained from lemon leaves: I—choline chloride–ethylene glycol (1:2) [21], II—triphenylmethylphosphonium bromide–ethylene glycol (1:5) [22], and III—choline chloride–glycerol (1:1.5) [23]. The effectiveness of these specific formulations is linked to the distinct nature of their components: choline chloride-based DESs are hydrophilic, with a molecular structure primarily stabilized by hydrogen bonding, while phosphonium-based DESs are hydrophobic, characterized by a structure dominated by a strong network of dispersive forces. Analyzing the behavior of these two different families of DESs can offer valuable insights for the development of more effective formulations.

GC chromatography coupled with MS spectrometry was used to evaluate the releasing performances of systems I–III, revealing distinct behavior in system III, composed of choline chloride–glycerol. This system proved to be more efficient in trapping the volatile mixture of the citrus leaf EO, providing its slower release. A sustainability and safety assessment was also conducted on the three systems, revealing the suitability of the choline chloride–glycerol system from both a sustainability and safety perspective.

## 2. Materials and Methods

### 2.1. Chemicals

Choline chloride (98%), methyltriphenyl phosphonium bromide (98%), ethylene glycol (99%), and glycerol (99%) were purchased by Merk Europe.

### 2.2. Essential Oil Production

Essential oil was obtained from 0.5 kg leaves from *Citrus limon* trees (“Tirso Agrumi” farm, located near Solarussa, Italy) suspended in 0.7 L of water and hydrodistilled for 2 h with a Clevenger-type apparatus [5]. The extraction was carried out in duplicate and the obtained essential oil (EO) was collected, dried over anhydrous Na_2_SO_4_, and stored at 4 °C under N_2_ in glass vials until analysis.

### 2.3. Eutectic Systems Preparation

Binary DESs were prepared on a 1 g scale by mixing the two components in a vial equipped with a magnetic stirrer. The mixture was then heated at 80 °C and stirred for 1 h.

#### 2.3.1. Static Headspace Extraction

In total, 200 mg of DESs containing 0.01 g (about 10% *w*/*w*) of EO in a 20 mL headspace vial, tightly closed with a septum, were extracted using an Agilent 7694E, Palo Alto, CA, USA, automatic autosampler for headspace analysis. The extraction of volatiles was carried out after equilibration at 60 °C whilst shaking for 3 min. Loop and transfer lines were maintained at 140 °C. Helium was used as the carrier gas and nitrogen as the pressurization gas.

#### 2.3.2. Gas Chromatograph–Mass Spectrometer (GC-MS) Analysis

Chemical analysis was carried out using an Agilent 6850 GC, Palo Alto, USA system coupled with an Agilent 5973 Mass Selective Detector, Palo Alto, USA. The chromatographic separation was performed on an HP-5 capillary column (30 m 0.25 mm, film thickness 0.17 μm). The following temperature program was used: 50 °C was maintained for 3 min, then increased to 210 °C at a rate of 4 °C/min, maintained for 15 min, and then increased at a rate of 10 °C/min up to 300 °C, which was finally maintained for 15 min. Helium was used as the carrier gas at a constant flow rate of 1 mL/min. Individual identification of the components was carried out by comparing the fragmentation spectra of the unknown molecules, separated into peaks by the GC component, with the spectra of known molecules available on the NIST online database. By comparing the retention indices of the unknown components of the EO that were experimentally detected with those of the NIST database, it was possible to further confirm the identification of the single molecules characterized by the fragmentation spectrum obtained by GC-MS. A solution of linear alkanes (C7–C22) was initially prepared and analyzed according to the same instrumental program applied for the EO samples in the same chromatographic column (HP-5), and Van den Dool and Kartz’s equation was applied in the calculation of the retention index (RI) [24].

## 3. Results and Discussion

### 3.1. Release Experiments

The following DESs were tested as solvents and releasing agents for citrus leaf EO (Table 2).

**Figure 1:**
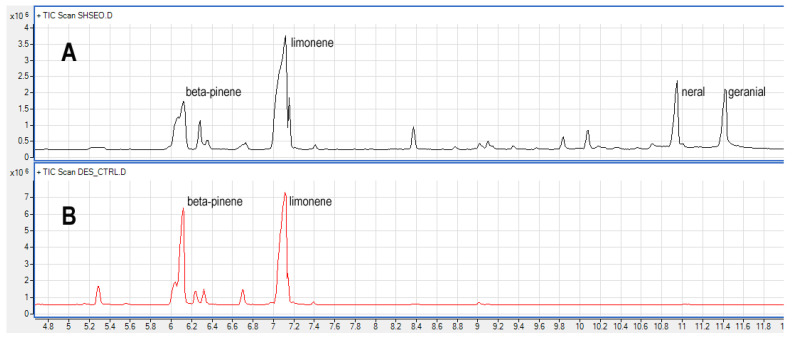
GC chromatogram of pure EO obtained by liquid injection (**A**), and headspace analysis (**B**).

According to previous studies [10], the GC-MS analysis of the pure EO herein studied revealed the presence, as the main constituent, of limonene, which dominates the chemical fingerprint, accounting for 38% of the whole chromatogram, followed by neral and geranial, which represent about 10% each of the chromatogram (Table 3). Besides the liquid injection of the raw essential oil, several experiments were carried out on the vapor fraction in the headspace. The direct comparison of the liquid injection vs headspace analysis of the same essential oil (Table 3, pure oil vs. CTRL) shows, as expected, that the headspace fraction is enriched in compounds with a higher vapor pressure, with respect to those with a lower one and, consequently, a higher boiling point. By looking at Figure 1, which represents the comparison of chromatograms of the same essential oil in the headspace and liquid injection, it is possible to observe an almost flat chromatogram in the headspace analysis after 7.6 min, supporting this conclusion.

**Figure 2:**
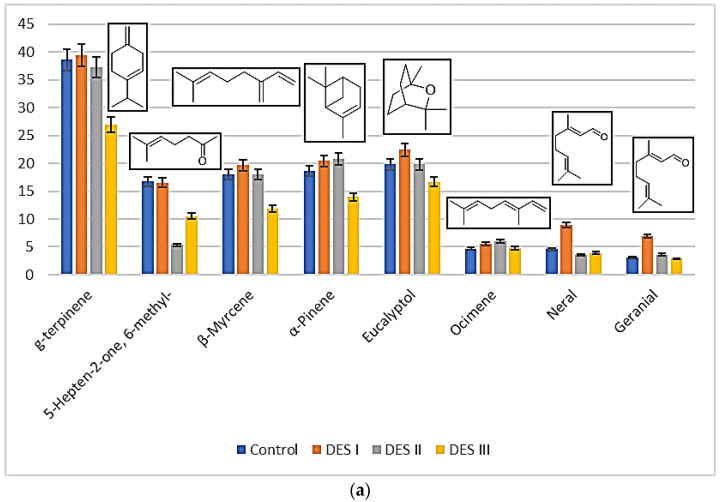

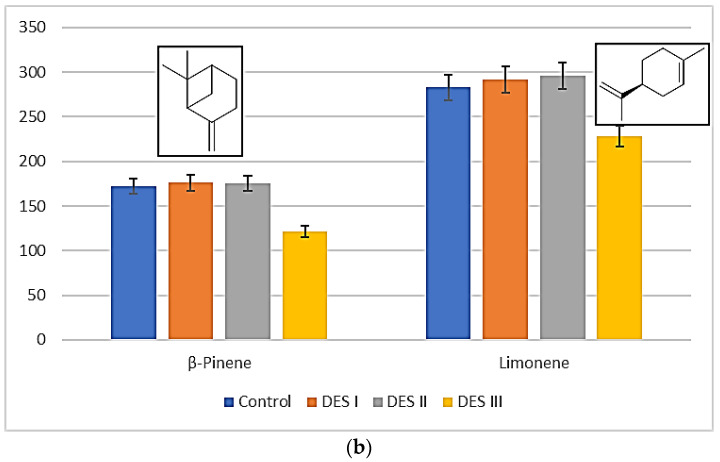
Releasing performance of systems I–III for VOCs expressed as absolute values of γ-terpinene, 5-Hepten-2-one, 6-methyl-, β-Myrcene, α-Pinene, eucalyptol, ocimene, neral, and geranial (**a**); β-pinene and limonene (**b**).

Examining the chemical characteristics of the main VOCs detected in the tested essential oil (EO), we can observe the presence of ketones that can interact with DESs through hydrogen bonding. Additionally, interactions involving other chemical components through dispersive forces cannot be excluded. A direct comparison of the interactions between DESs and essential oils was performed based on the absolute area of the chromatogram, as the VOC profile was assessed from the same amount of EO dissolved in DESs I, II, and III. The results are shown in Figure 2.

Looking at the data reported in Figure 1, it is possible to see that specific organic molecules can be released in a different way depending on the specific DES used. As a general trend, the EO dissolved in DES III (choline chloride–glycerol) released slower than all the VOCs considered with respect to the control experiments (first bar on the left and last on the right for each VOC, Figure 2). When DES I (choline chloride–ethylene glycol) was used, the volatility of the aldehydes neral and geranial resulted in incremented with respect to the other solvents, as well as to the control. This behavior suggests that system I is, in some way, able to disrupt the interactions occurring between the aldehydes and the other terpenes (mostly alkenes), making their release faster. This effect was not observed when glycerol, instead of ethylene glycol, was used as a hydrogen bond donor (DES III). Also, a peculiar behavior was detected for 5-Hepten-2-one, 6-methyl-, whose volatility was sensibly decreased when the EO was dissolved in DES II (methyltriphenylphosphonium bromide–glycerol). To the best of our knowledge, this ketone has not been previously detected in the leaves of *Citrus limon*. Its presence in the essential oil could be due to the degradation of citral aldehydes, as previously described in the literature [25]. It should be considered that DESs I and III are considered hydrophilic [26], with a molecular structure based mostly on hydrogen bonds, while the phosphonium DES II is hydrophobic [27], showing a consistent contribution to dispersive forces with respect to hydrogen bonds. The different nature and characteristics of the two types of DESs were then reflected by their behavior with the ketone 5-Hepten-2-one, 6-methyl-.

**Figure 3:**
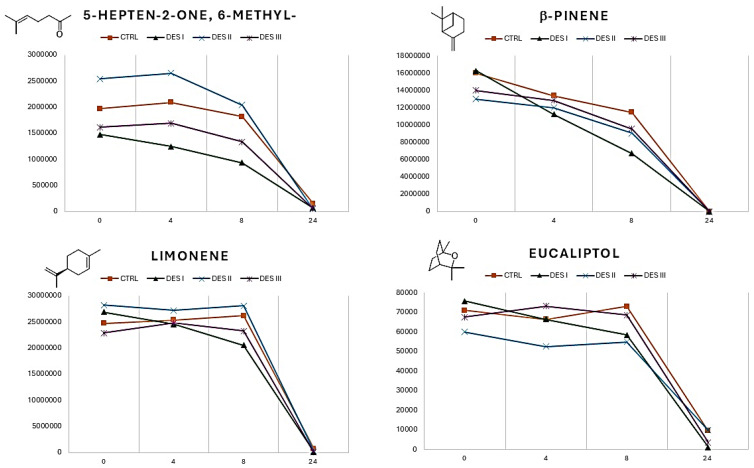
Variation in the VOCs 5-hepten-2-one, 6-methyl-, b-pinene, limonene, and eucalyptol versus the pure EO (CTRL) in the 24 h taken for the EO to dissolve in DESs I, II, and III. For each compound, the *y* and *x* axes report, respectively, the values of the absolute integrals and the time of analysis.

**Figure 4:**
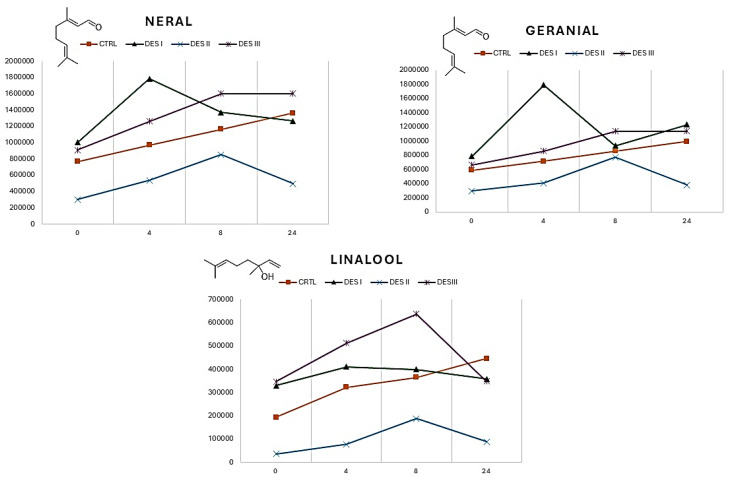
Variation in the VOCs neral, geranial, and linalool versus the pure EO (CTRL) in the 24 h taken for the EO to dissolve in DESs I, II, and III.

It is important to highlight that an EO is a complex mixture of chemicals whose physical characteristics, including volatility, result from various interactions and equilibria among its constituents, including the influence of synergistic effects. Eutectic solvents of a different nature (hydrophilic or hydrophobic) can interact differently with specific molecules. However, the primary trend observed is the significant decrease in the overall volatility of the essential oil when choline chloride–glycerol is used as the solvent. To provide additional evaluation data, the variation in the volatile profile (neral, geranial, eucaliptol, limonene, 5-Hepten-2-one, 6-methyl-, linalool, β-pinene) of the EO dissolved in the three DESs over 24 h was recorded (Figure 3 and Figure 4).

Looking at the plots reported in Figure 3, it is possible to highlight some differences within the volatility of the single VOCs when the EO is dissolved in the DESs (I, II, and III) or is analyzed as pure (CTRL curves). The monitoring of 5-hepten-2-one, 6-methyl- and limonene showed no significant deviations from the control samples. In the case of β-pinene, the releasing in DES I (choline chloride ethylene glycol) followed a different kinetic, with respect to the control, resulting in linear releasing during this time. Finally, DES II (methyltriphenylphosphonium bromide ethylene glycol) impacted the volatility of eucaliptol, which is lower than in pure EO. All the VOCs reported in Figure 3 show curves which end at the same point after 24 h. Bigger differences can be observed if the volatility of neral, geranial, and linalool is monitored (Figure 4).

For all the three VOCs reported in Figure 4, the pure EO shows a linear releasing over time, while the release kinetics of the DESs differ. It is important to note that neral and geranial are aldehydes, whereas linalool is an unsaturated alcohol, meaning all these VOCs have chemical groups sensitive to hydrogen bonding. A precise explanation of the release kinetics for individual VOCs would be unreliable due to the complex network of interactions that can occur within the EO and in the different DESs. However, certain trends can still be observed. When the EO is dissolved in DES II (methyltriphenylphosphoiunm bromide ethylene glycol), the volatility of both aldehyde and linalool is reduced compared to the control. Additionally, some volatile spikes can be seen, with an increase in neral and geranial in DES I, and in linalool in DES II. Finally, after 24 h, the volatile content of the VOCs reported in Figure 4 show notable differences, indicating that these polar molecules are highly sensitive to changes in the EO’s chemical composition.

To complete the assessment of the considered systems from a sustainability point of view, they were ranked according to the EcoScale procedure.

### 3.2. Sustainability and Safety Assessment

The analysis of the variation of the VOCs profile upon dissolution on systems I, II, and III showed a different behavior depending on the specific type of molecule to be released (aldehydes, ketones, alkenes), with DES III showing a distinctive ability to decrease the overall volatility of the EO. To also explore the environmental and safety impact of the three systems considered, especially in the view of the potential application of these systems in medicine or food devices, the EcoScale tool was used. According to Van Aken, sustainability and safety can be assessed by using the semiquantitative analysis EcoScale, which, starting from a score of 100, assigns penalty points to several aspects related to the sustainability and safety of the process (Equation (1)) [28].

EcoScale score = 100 − Σ individual penalty points(1)

Penalty points refer to yield, the price of the components, safety, technical setup, temperature, and workup [28].

The adaptation of the EcoScale to the three scenarios corresponding to the dissolution and releasing of the citrus leaf EO into DESs I, II, and III provided the following ranking (Table 4).

The original EcoScale was designed for chemical reactions and thus has been adapted to our scenarios, as they are simple from an operational point of view. The preparation of DESs was performed within a common laboratory setup (0 penalty points), at a temperature of 80 °C for more than 1 h (3 penalty points), and where no workup was necessary (0 penalty points). The parameter yield was related to the amount of citrus leaf EO that can be dissolved in the DES, and no differences were observed within the three systems, as they were able to quickly dissolve 10 mg of EO in 200 mg of DES (0 penalty points). The cost of the components was considered high (5 penalty points), as the obtention of 10 mg of EO was associated with an elevated cost. Although the same differences were present in the cost of the DESs’ components (with methyltriphenylphosphonium bromide being more expensive than choline chloride and the diols), the higher cost related to the EO pushed all three scenarios into the very expensive range.

What caused the real difference between scenarios I and III was the safety related to the components employed. Methyltriphenyl phosphonium bromide (CAS 1779-49-3) is classified as toxic if swallowed (H301) and toxic to aquatic life with long-lasting effects (H411), while ethylene glycol (CAS 107-21-1) is harmful if swallowed (H302) and may cause damage to organs (kidney) through prolonged or repeated exposure (if swallowed) (H373) [29]. On the other hand, choline chloride and glycerol are considered safe, making the DES composed of choline chloride and glycerol more suitable (97 EcoScale score versus 87 for the other scenarios), especially for application in medicine or food.

## 4. Conclusions

The potential of three deep eutectic solvents (DESs) as media for the controlled release of citrus leaf essential oil (EO) was explored. The EO was dissolved in choline chloride–ethlylene glycol, methyltriphenylphosphonium bromide–ethylene glycol, or choline chloride–glycerol, and the volatile fraction was analyzed through headspace gas chromatography. Two general trends emerged due to the nature of the volatile organic compounds (VOCs) present and their interactions with the three DESs. Choline-based DESs slowed the release of alkene VOCs, while enhancing the volatility of aldehyde-based neral and geranial. In contrast, phosphonium-based DES II significantly reduced the volatility of aldehyde VOCs and interacted poorly with less-polar terpenes. To assess which system is more suitable from a technical point of view, further studies should be conducted to determine the variation in the antimicrobial activity when polar terpenes (aldehydes or alcohols) are slowly released. Nevertheless, when sustainability and safety aspects are considered, system III (choline chloride–glycerol) should be preferred. In fact, an assessment of the sustainability and safety impact was conducted for the three scenarios using a penalty point system. As a result, an EcoScale was obtained, which indicates the choline chloride–glycerol system as the most suitable for practical industrial application, with a ranking of 97 points over 100 against the 87/100 attributed to systems I and II. In particular, the low toxicity of the choline chloride–glycerol system makes it suitable for incorporation into gels, meeting food-grade requirements for food packaging. Considering the parameters which determine the penalty points in the EcoScale, toxicity is the one which makes the difference. However, further studies are needed to enhance our understanding of long-term release kinetics, including comparisons between the release data of the present study (releasing of the individual VOCs in the dissolved EO) with the release performances of the single standards (without the matrix effect). Also, additional systems should be tested, after a screening based on sustainability performance, which can be conducted with the EcoScale penalty ranking tool.

## Figures and Tables

**Table 1:** Classification of most common DESs.

DES	General Formula
Type I	Cat^+^X^−^ · MCl_x_
Type II	Cat^+^X^−^ · MCl_x_ · yH_2_O
Type III	Cat^+^X^−^ · RZ
Type IV	MCl_x_ · RZ
Type V	non ionic

**Table 2:** DESs tested and their composition.

DESs (Molar Ratio)	Short Name	Components
Choline chloride–ethylene glycol (ChCl:EG 1:2)	I	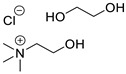
truphenylmetnhylphosphonium bromide–ethylene glycol (Ph_3_PMeBr:EG 1:5)	II	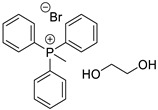
Choline chloride–glycerol (ChCl:GLY 1:1.5)	III	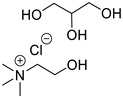

**Table 3:** Main composition of the volatile fraction of citrus leaf EO.

	RI	Pure EO	CTRL	SD	DES I	SD	DES II	SD	DES III	SD
γ-terpinene	976	6.81	6.41	0.66	6.24	0.17	6.11	0.89	5.82	0.48
β-Pinene	979	10.32	28.54	0.87	27.77	0.74	28.65	1.02	26.18	3.10
5-Hepten-2-one, 6-methyl-	988	3.32	2.78	0.03	2.62	0.14	0.88	0.09	2.27	0.09
β-Myrcene	992	1.21	2.98	0.32	3.11	0.06	2.95	0.24	2.56	0.08
α-Pinene	1012	1.64	3.09	0.05	3.22	0.05	3.40	0.09	3.02	0.03
Limonene	1033	38.08	46.85	0.91	46.06	1.30	48.35	0.37	49.44	2.33
Eucalyptol	1035	4.30	3.29	0.05	3.57	0.44	3.23	0.17	3.62	0.68
Ocimene	1048	1.00	0.77	0.39	0.90	0.54	1.00	0.40	1.03	0.28
Linalool	1100	2.59	tr	tr	tr	tr	tr	tr	tr	
Limonene oxide	1136	0.61	tr	tr	tr	tr	tr	tr	tr	
Limonene oxide	1140	0.69	tr	tr	tr	tr	tr	tr	tr	
Citronellal	1154	0.42	tr	tr	tr	tr	tr	tr	tr	
Terpinen-4-ol	1180	1.59	tr	tr	tr	tr	tr	tr	tr	
α-Terpineol	1194	2.40	tr	tr	tr	tr	tr	tr	tr	
Neral	1246	10.44	0.76	0.04	1.43	0.50	0.58	0.08	1.90	0.51
Geranial	1274	9.86	0.52	0.05	1.11	0.29	0.59	0.04	1.04	0.67
Neryl acetate	1358	2.85	tr	tr	tr	tr	tr	tr	tr	
Geranyl acetate	1383	1.87	tr	tr	tr	tr	tr	tr	tr	

Results are expressed as the means of three replicates of the relative percent obtained by the internal normalization of the chromatogram. RI: the calculated retention index on an HP5 column. SD: standard deviation. CTRL stands for “control”, meaning that the EO was analyzed in its pure form. tr stands for traces. DESs I, II, and III are reported in Table 1.

**Table 4:** Calculation of EcoScale score for scenarios 1 (choline chloride–ethylene glycol), 2 (methyltriphenylphosphonium bromide–ethylene glycol), and 3 (choline chloride–glycerol). Each column reports the corresponding penalty points calculated with the EcoScale of Van Aken [28].

Parameter	Scenario 1 (Penalty Points)	Scenario 2 (Penalty Points)	Scenario 3 (Penalty Points)
1. Yield	0	0	0
2. Price of components	5	5	5
3. Safety	10	10	0
4. Technical setup	0	0	0
5. Temperature	3	3	3
6. Workup	0	0	0
EcoScale score	87	87	97

## Data Availability

The original contributions presented in this study are included in this article, and further inquiries can be directed to the corresponding author.
